# Social Cognition for Human-Robot Symbiosis—Challenges and Building Blocks

**DOI:** 10.3389/fnbot.2018.00034

**Published:** 2018-07-11

**Authors:** Giulio Sandini, Vishwanathan Mohan, Alessandra Sciutti, Pietro Morasso

**Affiliations:** ^1^Research Unit of Robotics, Brain, and Cognitive Sciences (RBCS), Istituto Italiano di Tecnologia, Genoa, Italy; ^2^School of Computer Science and Electronic Engineering, University of Essex, Colchester, United Kingdom

**Keywords:** cognitive architecture, social interaction, human robot symbiosis, memory system, internal model, cognitive robotics, imitation, lifelong learning

## Abstract

The next generation of robot companions or robot working partners will need to satisfy social requirements somehow similar to the famous laws of robotics envisaged by Isaac Asimov time ago (Asimov, 1942). The necessary technology has almost reached the required level, including sensors and actuators, but the cognitive organization is still in its infancy and is only partially supported by the current understanding of brain cognitive processes. The brain of symbiotic robots will certainly not be a “positronic” replica of the human brain: probably, the greatest part of it will be a set of interacting computational processes running in the cloud. In this article, we review the challenges that must be met in the design of a set of interacting computational processes as building blocks of a cognitive architecture that may give symbiotic capabilities to collaborative robots of the next decades: (1) an animated body-schema; (2) an imitation machinery; (3) a motor intentions machinery; (4) a set of physical interaction mechanisms; and (5) a shared memory system for incremental symbiotic development. We would like to stress that our approach is totally un-hierarchical: the five building blocks of the shared cognitive architecture are fully bi-directionally connected. For example, imitation and intentional processes require the “services” of the animated body schema which, on the other hand, can run its simulations if appropriately prompted by imitation and/or intention, with or without physical interaction. Successful experiences can leave a trace in the shared memory system and chunks of memory fragment may compete to participate to novel cooperative actions. And so on and so forth. At the heart of the system is lifelong training and learning but, different from the conventional learning paradigms in neural networks, where learning is somehow passively imposed by an external agent, in symbiotic robots there is an element of free choice of what is worth learning, driven by the interaction between the robot and the human partner. The proposed set of building blocks is certainly a rough approximation of what is needed by symbiotic robots but we believe it is a useful starting point for building a computational framework.

## Introduction

Symbiosis: what does it mean, exactly? Although this question has been the topic of debate for over a century, current biology and ecology textbooks generally agree to use an early and rather broad definition proposed by the German biologist Heinrich Anton de Bary at the end of the 19th century: “Symbiosis is the living together of unlike organisms.” Usually, this definition takes for granted that both organisms are biological. Licklider (1960) was probably the first one to extend the paradigm, assuming that one element of the pair could be cybernetic instead of biological. At that time, the cybernetic organism was a computer and the communication with the human organism was mainly restricted to language. As a matter of fact, Licklider was well aware that one of the crucially limiting aspects for an effective human-computer symbiosis was the inadequacy of “input-output” devices. Nowadays, a computer with a rich set of input-output devices is a robot, with the crucial challenge of integrating multi-sensory perception, skilled motor control and cognitive capabilities.

Although robotics is a highly differentiated technological/scientific area, there is no doubt that industrial robotics, namely the application of robotics to manufacturing, has driven the development and accumulation of knowledge of the whole field. Industrial robotics took off in the early 60’s and since then expanded steadily, supporting the third wave of the industrial revolution, characterized indeed by an increasing degree of automatization/robotization. However, this process did not include any element of human-robot symbiosis: robots were restricted behind “cages” as a solution to safe operation of robots repeating endlessly operational sequences explicitly avoiding any kind of bidirectional interaction and coded once for all by human operators. On the other hand, the next wave of the industrial revolution (industry 4.0) has all the features of a paradigm shift in general, with specific effects in the case of robotics (Hermann et al., 2016) that will allow robots, as cyber-physical systems, to get out of the “cages” and interact safely with human colleagues as cooperating robots or “cobots.” This will require to trade-off speed and positional accuracy (the main goal of human-less robotics) with time-scales that are consistent with human behavior, focusing also on what has been defined as “social moments” of human-robot interaction (Durantin et al., 2017). It is also expected that such evolution will be characterized, in the initial phase, by improving sensitive technologies capable to increase the safety of human-robot interaction; this essential milestone will be accompanied by an increasing level of mobility for allowing human-robot cooperation in cluttered workspaces, with the final goal of achieving intelligent cobot technologies with full cognitive capabilities. There is also no doubt that such quantum changes of the next wave of robots will spread beyond the manufacturing sector, allowing humans to interact with robots physically and cognitively in many ways, at many levels, and in many application areas, including social applications related to health and the aging society.

For an effective, successful, and safe support of humans in everyday tasks, it is necessary that humans can interact smoothly and intuitively with their robotic partners, also through a proper training for this kind of human-machine collaboration. However, while we can be confident that incremental technological advances will pave the road for achieving the necessary improvements in cobot technologies (in terms of power/energy, material properties, sensing, actuation, mobility and manipulation capabilities), the issues of cognitive capabilities are quite another type of challenge, characterized by scientific as well as technological aspects that are far from being sufficiently understood for symbiotic robotic agents. The literature on cognitive architectures is quite large and with a long history, mixed with the general issues of artificial intelligence (Newell, 1990). Among the many proposals of Cognitivist, Emergent and Hybrid frameworks (see Kumova and Heye, 2017; Lieto et al., 2018, for a review), some have been implemented on various robots to facilitate problem solving, cumulative learning, goal directed reasoning and communication. For example, agent architectures such as ACT-R (Anderson, 1996) and Soar (Laird, 2012) deploy highly parallel, asynchronous processing and rely on symbolic rule-based modules to provide global control. Presently, attempts are ongoing to integrate well known frameworks like ACT-R, SOAR and SIGMA into a unified standard model of cognition (Laird et al., 2017). However, as these authors point out, while the standard model brings about a convergence in several aspects of cognition, it is incomplete in the context of mental imagery, metacognition, communication and mechanisms needed for social cognition.

This article is a contribution on this topic, based on the assumption that human robot-symbiosis is fundamentally a problem of cognition, more specifically social cognition, that addresses cognitive issues related to the interaction of individuals in a society. For simplicity, we will keep out of our analysis issues of affective interaction (de Gelder, 2006; Duffy, 2008; Breazeal, 2009; Wiese et al., 2017), although empathy contributes to efficient interactions and is obviously relevant for inducing humans to eventually accept robots as social companions. On this purpose, we will focus on fundamental building blocks for allowing a robot to physically interact with a human in a symbiotic manner, at the same time amenable to further extensions in the direction of affective interaction, for example a grounded taxonomy of emotional affordances (Vallverdú and Trovato, 2016). We wish also to emphasize that the overall approach fully agrees with the general principles of *embodied cognition* (Wilson, 2002; Pfeifer, 2006; Pfeifer et al., 2008; Vernon et al., 2015a,b), which link together mental processes with physical interaction processes: the motor system may indeed influence cognitive states and the latter may affect bodily actions. In particular, in our context the main features of embodied cognition can be summarized as follows: (1) *cognition is situated*, namely it is an online process which takes place in the context of task-relevant sensorimotor information; (2) *cognition is time pressured*, because it is constrained by the requirements of physical real-time interactions; (3) *cognition is ecologic*, in the sense that the environment is part of the cognitive system, including both the physical and social environment; (4) *cognition is intrinsically action oriented* and even “off-line cognition,” namely cognition without overt action, is bodily based. Moreover, the well-established reciprocal coupling of perception and action in cognitive agents can be characterized by what has been described as “circular causality” (Vernon et al., 2015a) which considers explicitly motivations and expectations.

Also on the human side, we need to restrict our focus in order to outline a computational framework for human-robot symbiosis. As a matter of fact, social cognition is a sub-topic of social psychology that focuses on how people process, store, and apply information about other people and social situations and thus it is concerned with the role that cognitive processes play in social interactions. How relevant may this be for human-robot symbiosis? Think of artificial intelligence: the idea that in order to achieve intelligent behavior in cybernetic systems one needs to be inspired specifically by human intelligence has been strongly challenged in the past and the new wave of intelligent artifacts, driven by machine learning methods, is indeed quite detached from cognitive neuroscience (Michalski et al., 1984; Arel et al., 2010; Jordan and Mitchell, 2015). But in the case of human-robot symbiosis such disconnection is totally inappropriate. Symbiosis requires not only channels of explicit/implicit communication, but also the capability to access and/or anticipate the partner’s internal states and intentions during a cooperative task: for example, a verbal exchange may be sufficient to identify the goal of a shared task but the skilled sequence of actions that follow require a deep mutual understanding of what is going on, including both overt and covert actions of both partners. This means that a symbiotic robot must share with human partners an overall cognitive architecture, although the detailed implementation may be different and the technologies adopted will certainly be different, possibly substituting the current hardware—silicon and metal—in terms of wetware.

## Basic Elements of a Shared Cognitive Architecture for Human-Robot Symbiosis

In the following paragraphs, we outline what we think are basic elements of such architecture: (1) an animated body schema; (2) an imitation mechanism; (3) motor intention machinery; (4) physical interaction paradigms for skill transfer; and (5) a shared memory system for incremental symbiotic development, including a shared model of each other. Such building blocks are not independent but are characterized by intra and inter bidirectional interactions: inside the mind of each cooperating partner or also across their minds, with reference to visible (overt) actions as well as mental (covert) actions. Figure 8, that is discussed in depths in the concluding section of the article together with the associated Figure 9, is a graphical outline of the architecture.

**Figure 1:**
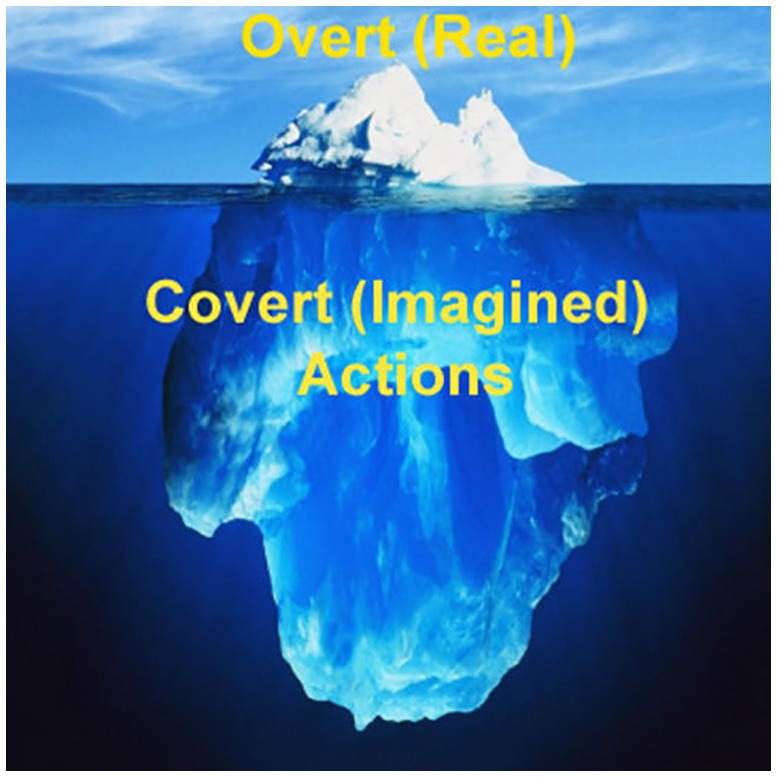
Overt vs. Covert actions: the real/overt actions are like the tip of an Iceberg: the great majority are apparently silent but effectively determinant.

**Figure 2:**
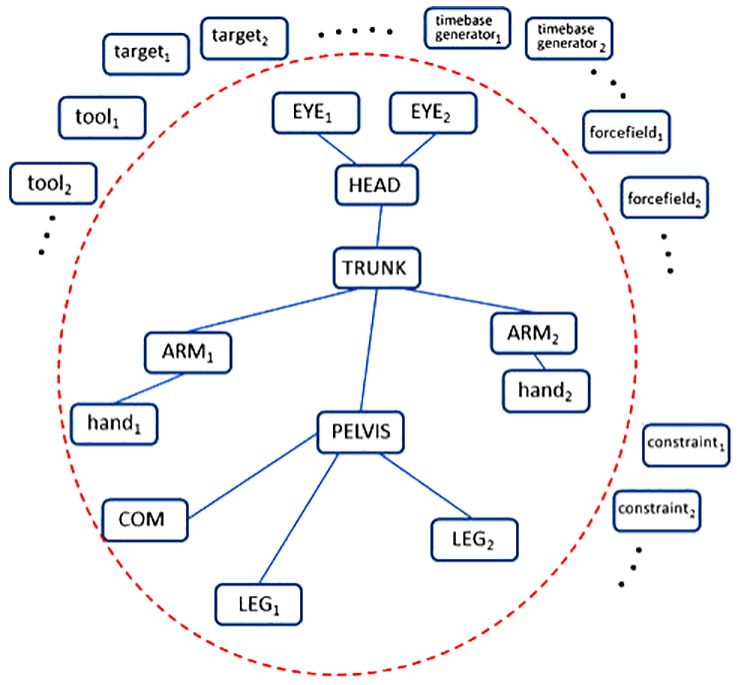
Animation of the body schema. The somatotopically organized body schema is dynamically linked to task-specific elements such as targets, tools, force fields and time base generators, thus breaking an equilibrium state and prompting the evolution to a new equilibrium, namely carrying out synergy formation. From Morasso et al. (2015).

**Figure 3:**
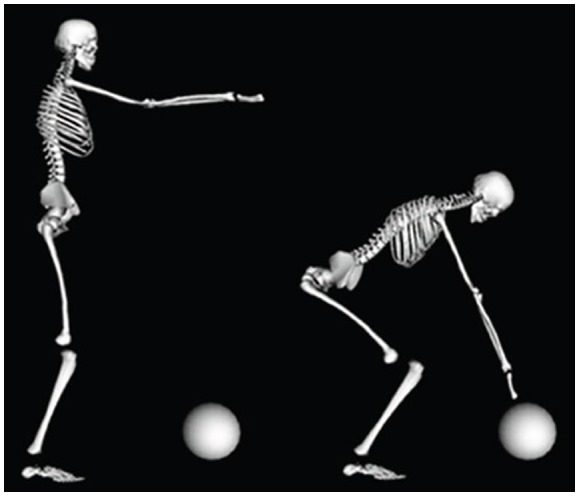
Initial and final frame of a whole-body reaching task obtained through the animation of the body schema by means of five concurrent force fields: (1) a Focal field, applied to the hand and pulling it to the designated target; (2) a Postural field, applied to the pelvis, that aims at keeping the projection of the CoM inside the support base; (3) a Range of Motion (ROM) field, applied to each joint in order to repulse joint angles from the physiological joint limits; (4) Head gaze field, that aims at keeping the gaze directed to the target by inducing appropriate rotations of the cervical joint; and (5) Neck field, that stabilizes the neck, by attempting to keep it approximately vertical. From Morasso et al. (2015).

**Figure 4:**
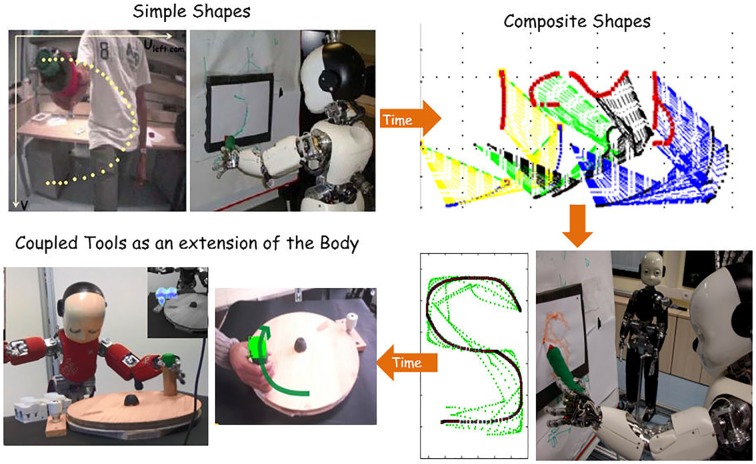
Imitation: a human teacher shows a humanoid robot (iCub) to draw “Shapes.” Unpublished picture, related to Mohan et al. (2011). Learning is cumulative and open ended several demonstrations of increasing complexity, so as to facilitate both “reuse” of past motor knowledge in new contexts and “compositionality.”

**Figure 5:**
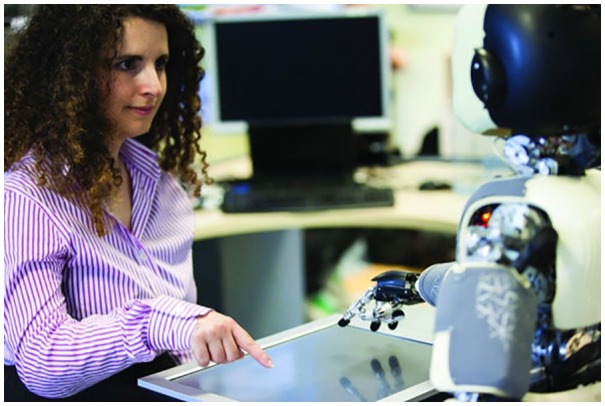
An illustrative picture of human-robot interaction. The mutual and spontaneous information exchange is mediated by context (i.e., the game on the touch screen that the two partners are playing) and by the agents’ gazing behavior, but also by the intention information embedded in their movement properties. From Sciutti et al. (2015).

**Figure 6:**
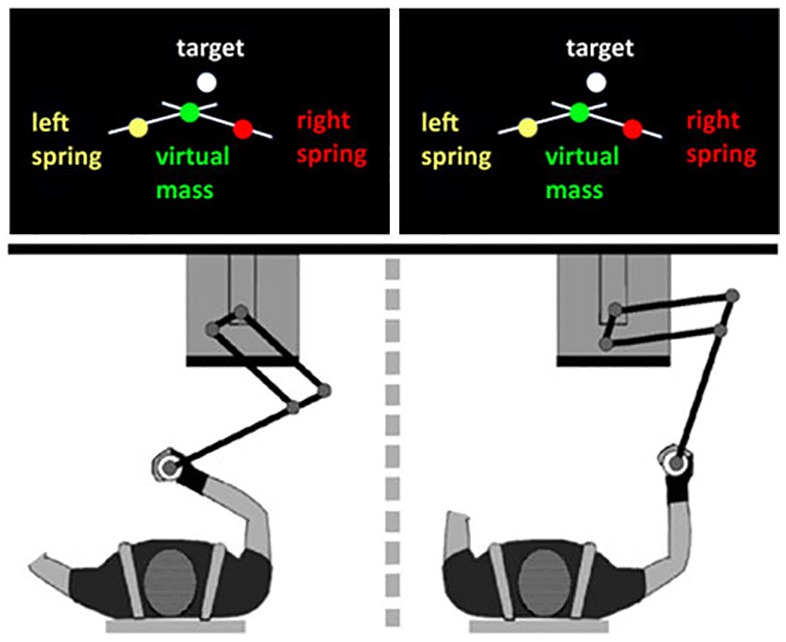
Dyadic interaction task consisting of reaching movements of a virtual device in an unstable dynamic environment using a bilateral non-linear elastic tool that could be used bimanually or dyadically, From Avila Mireles et al. (2017).

**Figure 7:**
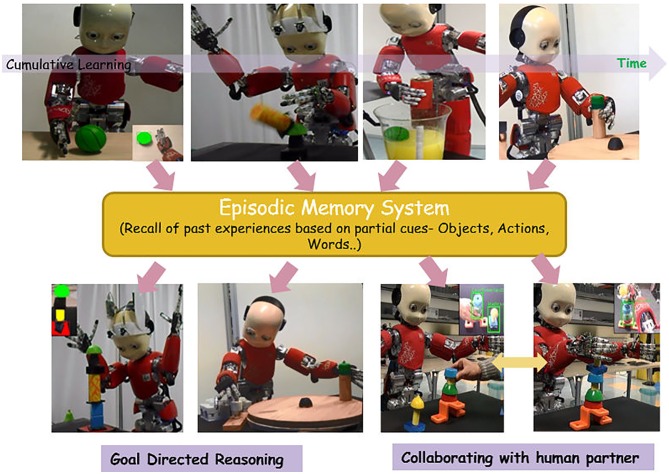
Cumulative learning and encoding of diverse experiences into iCub’s episodic memory. Unpublished picture, related to the article by Mohan et al. (2014) and Bhat et al. (2016). Diverse partial cues from the present environment (objects in the scene, observed actions of human counterpart, linguistic words like “Mushroom”, “Stack”, “Assemble”) trigger associative recall of past experiences that are subsequently used for both goal directed reasoning and joint goal human robot collaboration.

**Figure 8:**
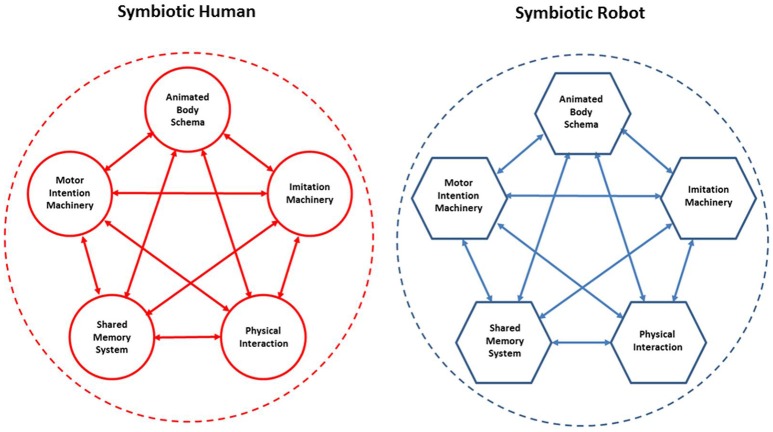
Building blocks for a symbiotic cognitive architecture shared by human and robot. The blocks are the same at a sufficiently high computational level, although the specific implementation details are likely to be quite different: this difference is iconically highlighted by the different shapes of the blocks (round vs. polygonal).

**Figure 9:**
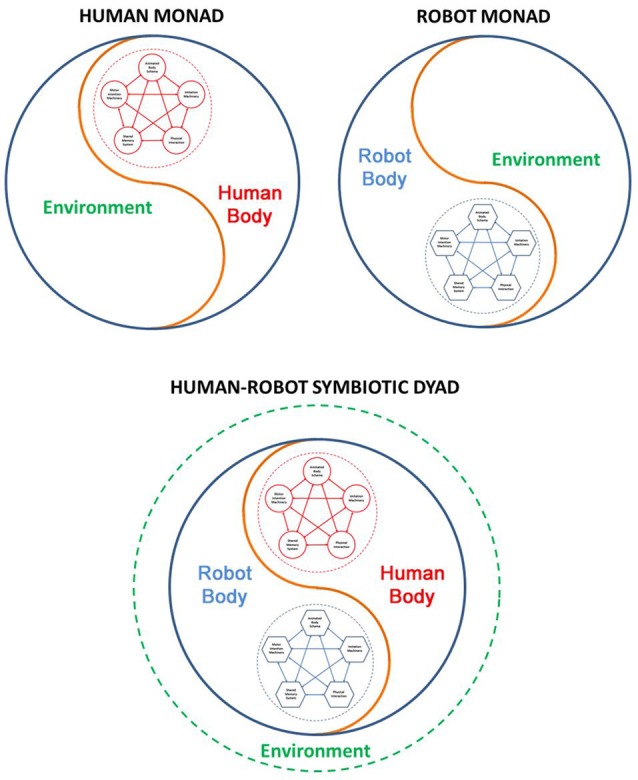
Embodied cognition in symbiotic human-robot interaction.

In general, symbiotic collaboration of robots and humans requires the integration of the individual partner’s intentions into a shared action plan, which may involve continuous negotiation of intentions based on reciprocal understanding of actions. There is ground to think that this process of intention integration is further enhanced via both auditory and haptic channels, which mainly operate in real-time via overt coordinated actions: consider, for example, “haptic dancing” (Gentry and Murray-Smith, 2003; Wang and Kosuge, 2012) where the communication between the partners is mediated mainly by this modality. Although this is a necessary requirement for successful collaboration, it is not sufficient, because it must be supported by a kind of resonance among the covert/mental actions of the partners, which prepare off-line their motor plans. In other words, symbiosis occurs with different time scales and different time horizons, in “real” time and “virtual” time, with an alternation of visible actions and mental actions similar to sound and silence in symphonic compositions.

In recent years, the investigation of motor cognition has focused on brain activity related to actions in the absence of movement and muscle activation, what we may call motion-less and muscle-less actions (Mohan et al., 2018). In particular, there is mounting evidence that cortical networks in the motor areas are activated in several contexts that do not involve overt movement, for example predicting consequences of potential actions during goal directed reasoning, observing/understanding others actions during social interactions (Gallese and Sinigaglia, 2011; Mohan et al., 2011; Rizzolatti and Fogassi, 2014). Undoubtedly, cobots assisting humans in diverse natural living spaces must be endowed with similar capabilities. In literature, such processes are understood within the frameworks of simulation theory (Jeannerod, 2001) or emulation theory (Grush, 2004; Ptak et al., 2017). Emulation theory suggests that emulation creates and maintains an abstract representation of movement kinematics over short time periods that can be manipulated to support motor imagery, both self-initiated and externally triggered. An emulator typically receives input from motor planning and outputs the sensory consequences as though the movement has been executed. Simulation theory (in the context of motor cognition) postulates that imagined actions rely on the same set of neural mechanisms as the real action, hence encapsulating the goal, the motor plan and sensory consequences, with the execution being inhibited during motor imagery. In this context, observing someone else “grasping an apple” or understanding the sentence “grasp an apple” entails a simulation recruiting the same motor areas that are also involved when one actually “grasps an apple.” While simulation and emulation are related and sometimes even used interchangeably, it is out of scope of this article to go into precise definitions and interpretations (see O’Shea and Moran, 2017 for a review). Instead, our focus is on the design of computational building blocks (inspired by ideas of emulation and simulation of actions) that would support human robot symbiosis. In cognitive robotics, while there is general consensus on the role of forward models for enabling action generation/simulation, the underlying computational formulations are based on a variety of paradigms such as optimal feedback control (Franklin and Wolpert, 2011), active inference based integral forward models (Friston, 2011), or body schema-based action generation/simulation networks (Mohan and Morasso, 2011; Bhat et al., 2016). Such forward models generally operate in a short time scale, for example predicting how to turn a handle to open the door or inferring the use of a tool (as an extension to the end effector) to reach an otherwise unreachable object. The crucial feature, irrespective of the underlying computational formulation, is “cognitive economy,” in the sense of allowing the reuse of a same system (in this case, action generation) to support other functions (action imagination, understanding)—which is highly relevant both in the context of the brain or an autonomous robot.

Simulation, however, is also used in a broader multi-modal sense (not just motoric), involving simulation of perception (see, Hesslow, 2012; Mohan et al., 2013; Martin, 2016) and requiring associative mechanisms that enable simulated perception and action to elicit each other, thus leading to inferences in longer time scale and involving long term memory of the agent (robot/human). Such mechanisms are referred to as constructive episodic simulation (Addis et al., 2011) and known to involve a core network in the brain (Buckner and Carroll, 2007; Benoit and Schacter, 2015; Schacter and Madore, 2016) that supports diverse functions like recalling the past, simulating future states/alternatives to events in the immediate environment, and inferring perspective of the other. For example, (Donoso et al., 2014) review the involvement of various prefrontal cortical areas to support reasoning and propose a Bayesian probabilistic framework operating over behavioral strategies stored in long-term memory for monitoring goals, switching strategies and learning new ones (as necessary). Mohan et al. (2014) proposed a neural episodic memory architecture for robots enabling multiple functions like recalling past experiences (based on environmental cues—objects in the environment), simulation of future states with potential, ensuing rewards and inferring goal of the user (in which case, the observed actions act as a partial cue triggering episodic simulation). Such processes related to episodic simulation and perspective taking are also highly relevant while considering long-term engagement of human and robot in any shared working environment (domestic or industrial). In the next subsections, we explore some of the core subsystems and their relevance to human robot symbiosis.

## An Animated Body-Schema

An animated body-schema is a fundamental building block of the envisaged cognitive architecture for symbiotic human-robot symbiotic interaction. Any simple action, even the apparently simplest one, potentially involves the full complexity of the human body. For example, the task of reaching a target with your fingertip directly involves the coordination of only two joints, by activating the corresponding muscle groups, but the performance of this task generates internal disturbances to the posture of the body that need to be compensated by modification of the muscle activities of other body parts, e.g., trunk and legs if the subject is standing on one or two legs. In order to tackle such overwhelming task, the human brain has to find a trade-off between two contrasting requirements, exemplified by Bernstein’s *Degrees of Freedom Problem* and Lashley’s *Principle of Motor Equivalence* (Lashley, 1933; Bernstein, 1935). In this context, an underlying deeper question raised by different authors (Friston, 2011; Mohan and Morasso, 2011; Herbort and Butz, 2012; Pickering and Clark, 2014) can be formulated as follows: in purposive actions, is it more appropriate to conceive that muscle activations “cause” joint rotations that “cause” the end-effector motion or the other way round? The animated body-schema that we are proposing as one of the fundamental building blocks of *symbiotic robots* totally agrees with the latter alternative: it is an integrated internal sensory-motor dynamic model (Morasso et al., 2015) driven by intentionality and characterized by three main characteristics, namely *Multireferentiality*, *Plasticity* and *Virtuality* as described in the next three subsections.

### Multireferentiality

The principle of *multireferentiality* goes against the common wisdom that specific experimental paradigms are better explained with a frame of reference or another, e.g., extrinsic Cartesian frames of reference associated with end-effectors or intrinsic reference frames, joint or muscle oriented. One of the main problems faced by the brain is indeed to maintain the coherence among spatial information in cortical maps and fronto-parietal circuits for controlling body movements in a large variety of tasks (Haggard and Wolpert, 2005). In a study that systematically investigated a large workspace for reaching movements, solid evidence was found against a single coordinate system representation in the motor cortex, thus suggesting the possible coexistence of multiple reference frames (Wu and Hatsopoulos, 2006). Moreover, the observed heterogeneity and ambiguity of neuronal responses (Churchland and Shenoy, 2007) suggested that it may be necessary to discard the notion that the key to understand the organization of sensorimotor cortical areas is to pursue the search of the code of movement parameters representation.

From the mathematical point of view, we may conceive the body schema as a collection of Jacobian matrices, i.e., simple operators that link extrinsic and intrinsic variables of the different body parts, thus allowing the coexistence of multi-referential representations. Such ensemble of Jacobian operators is somatotopically organized in a body-network that replicates the body structure. Moreover, the body schema is not a static internal model that simply reflects, as in the classical homunculus metaphor, the motor/perceptual activity of different body parts: on the contrary, it is a dynamic internal model that needs to be “animated” by a set of task-related and environment-related mechanisms that, as better explained in the following, can be conceived as virtual force fields. In short, the animation of the body schema is equivalent to the simulation of this internal model. The Jacobian matrices that constitute the core of the body schema can be evaluated by means of a variety of techniques of babbling and self-observation/self-analysis (Hersch et al., 2008; Sturm et al., 2009; Mohan et al., 2013).

### Plasticity

The *plasticity* of the body schema refers to three types of relevant phenomena:

1. The human/robotic body can change its geometry during development or other occurrences such as aging, fatigue, or wearing personal protective equipment (PPE) like fire protection suits or heavy loads and the corresponding body schema must also adapt to such changes;
2. The human/robotic body-schema can be extended by the use of tools (Maravita and Iriki, 2004; Iriki and Sakura, 2008; Umiltà et al., 2008) and we may assume that the corresponding Jacobians are learned during training for mastering the tool use;
3. When observing/reasoning about the action of a cooperating partner a human or humanoid robotic operator may need to duplicate his own body schema in order to approximate the actions of the partner; as further speculated later on, this may also be one of the mechanisms of imitation. Interacting with a child or an elderly activates different body schemas.

The learning techniques already mentioned for acquiring the mathematical model of the body schema can be applied as well for adapting it to such kind of changing conditions, showing also the reciprocity of learning to act and to understand.

### Virtuality

The *virtuality* of the body schema is intended to capture the concept that the body schema should operate equally well and in the same manner for real or overt actions as well as for imagined or covert actions. This is very important because during any goal oriented and/or social behavior we are both acting and anticipating the consequences, feasibility, goals of “potential actions” of oneself or other. In this context, overt actions are just the tip of the iceberg (Figure 1) and in the concept of virtuality is a basic requirement for human-robot symbiosis. As a consequence, the body schema by itself can be conceived as a mass-less/muscle-less entity because covert actions cannot involve, directly masses/loads/contraction of muscles. However, when the simulation of the body schema drives the execution of overt movement it must interact with another computational layer related to the real-time control of muscles and masses. This is the classical distinction between kinematics and dynamics. In conventional robotics, which ignores the complementarity of overt and covert actions, dynamics is hierarchically on a higher level than kinematics/geometry. In our framework kinematics and dynamics are, again, two faces of the same coin, without any hierarchical relationship. A computational metaphor, for explaining this kind of relationship, is captured by Disney’s animated character Peter Pan, with the female protagonist Wendy Darling sewing his shadow back to his body as a necessary prerequisite for freely and skillfully acting. Similarly, it is crucial for a symbiotic robot to connect its “metal and wire” body with its ubiquitous shadow (i.e., its internal representation of its body).

#### Animation of the Body Schema

The *animation* of the body schema is carried out by means of virtual force fields. Consider that Jacobian matrices operate in a bilateral manner: in one direction they map incremental motion from one (proximal) space to another (distal) space, following the direction of causality (e.g., from the joint space to the end-effector space of the arm); in the opposite direction, the same (transpose) Jacobian maps a virtual force (e.g., applied to the end-effector of the arm) into an equivalent virtual torque (applied to the joints of the arm), thus implementing the equilibrium principle of virtual works. To summarize, the body network is a graph with two types of nodes (force-like and position-like) and two types of connections (geometric-like, i.e., Jacobian matrices, and elastic-like, expressing local compliance to a virtual force). Starting from an initial equilibrium state, the animation is prompted by the activation of the force fields that disrupt equilibrium by propagating throughout the network until a new equilibrium is eventually reached. At the same time, incremental motions are propagated in the opposite direction, generating a complex coordinated global motion, thus carrying out a function of synergy formation. We wish to emphasize that this process operates with a high degree of motor redundancy, using only well posed transformations (Mohan and Morasso, 2011). Operating only through well posed transformations importantly offers computational simplicity by avoiding the need to choose one solution from many through a computationally expensive optimization process that can be prohibitive both for a complex humanoid or human. Formulations of forward models of action emerging from predictive coding and active inference (Friston, 2011; Pickering and Clark, 2014) also share similar properties of well posed transformations (circumventing the need for kinematic inversions). We believe that the body schema formulation is at the same time closer to bio mechanics (as an extension of the Equilibrium point hypothesis) and embodied cognition (Gallese and Sinigaglia, 2011; Clark, 2016; Ptak et al., 2017) in comparison to a more general Active Inference formulation.

The biological plausibility of a force-field based explanation of synergy formation has a long history, starting with the Equilibrium-Point Hypothesis (EPH: Bernstein, 1967; Asatryan and Feldman, 1965; Bizzi et al., 1976). The importance of force fields was also clarified in experiments on force-field adaptation, initiated by the seminal work of Shadmehr and Mussa-Ivaldi (1994). The EPH was extended in such a way to explain not only the synergy formation of real movements but also covert actions. The idea is known as Passive Motion Paradigm (PMP) and it has been developed over the years (Mussa Ivaldi et al., 1988; Morasso, 1992; Morasso and Sanguineti, 1995; Tsuji et al., 2002; Mohan and Morasso, 2007; Mohan et al., 2011, 2009, 2013; Bhat et al., 2016). According to this view, the coordination of a redundant set of degrees of freedom (DoFs) can be expressed by the relaxation of the kinematic network of the body to a force field that attracts the designated end-effector to a target. The key point is that in the animation of the action it is not the proximal part of the body which is pushing the end-effector to the target, but the other way around: the end-effector is pulled towards the target by the force field and in turn pulls the rest of the body in a coordinated fashion, in spite of the high degree of redundancy of the body schema. This idea can be further generalized in space and time:

1. *There is no reason for limiting the animation of the body-schema to a single force field*. Multiple force fields can be activated simultaneously, representing different tasks and different internal and external constraints that must be satisfied at the same time.
2. *There is also no reason for limiting the animation to single, isolated actions*. In everyday life purposive actions are generally composed of a smooth concatenation of primitive actions. For example, graphemes in the case of cursive handwriting (Morasso and Mussa Ivaldi, 1982) or phonemes in the case of connected speech (Clark and Yallop, 1995). The single primitive actions in a sequence are modulated/modified by the neighboring primitives. In the case of speech this phenomenon is named *coarticulation* and it refers to a situation in which a conceptually isolated speech sound is influenced by, and becomes more like, a preceding or following speech sound.

The biological plausibility of a force-field based formulation of the body-schema animation process was provided by Jeannerod (2001) who formulated the Mental Simulation Theory. This theory, supported by neuroscientific evidence, posits that cognitive motor processes such as motor imagery, movement observation, action planning and verbalization share the same representations with motor execution and are implemented by running an internal model of the body schema. Jeannerod interpreted this brain activity as an internal simulation of a detailed representation of action and used the term S-state for describing the corresponding time-varying mental states. The crucial point is that since S-states occurring during covert actions are, to a great extent, quite similar to the states occurring during overt actions, then it is not unreasonable to posit that also real, overt actions are the results of the same internal simulation process, operating on an internal schematization of the body, i.e., a body-schema.

The great computational power of a force-field based animation of the body schema is *compositionality*: superimposing, in space and time, multiple primitive force fields that express functional aspects of a given purposive action will generally produce a flow of coherent motor patterns. This would not be the case if the primitive elements were expressed in terms of movements, not force fields. For example, in the case of speech, coarticulation is a simple side effect of the force-field based animation without any need of specific rules or mechanisms for implementing the seamless transition from one phoneme to the next one. The same observation applies to cursive script, smoothly changing the shape of subsequent pen-strokes as a function of the preceding and the following ones. Compositionality is clearly essential for human/robot symbiosis. Moreover, the force-field based animation is *computationally simple* because the number of force fields required for a given task is generally small, compared to the overall complexity of the body, and smaller the simpler the task.

From the mathematical point of view, let $\vec{s}\in R^{n}$ be the state vector that identifies the body schema: *n* is the total number of degrees of freedom. We may conceive the animation of the body schema as the relaxation of the state vector to a bunch of force fields, say *m* of them, each of them defined in a reference frame *R*_*i*_, with the corresponding Jacobian matrix *J*_*i*_. Figure 2 illustrates the animation process: the somatotopically organized body schema (the “bunch” of Jacobian matrices) is dynamically linked to task-specific elements such as targets, tools, force fields, and time base generators, thus breaking an equilibrium state and prompting the evolution to a new equilibrium, namely carrying out synergy formation.

The preliminary step is retrieving from memory the force fields that are relevant for the given task and preparing the animation process by fine tuning the fields parameters:

(1) $$\begin{array}{ll} F_{i}^{R_{i}}=f_{i}(p_{i},\vec{s},t),\;i=1,\ldots m & \text{ Force filed selection and preparation} \end{array}$$

Here *f*_*i*_ is the specific force field generator that is a function of the state $\vec{s}$, time *t*, and the specific force field parameter vector *p*_*i*_. Of course, this memory retrieval operation is somehow subjective, in the sense of being driven by task-related attention to objects, tools, body parts of the partner, etc., and we may think of gaze as well as other body gestures as possible indexing elements for retrieval/rearrangement of the appropriate force fields. For animation the different force fields are superimposed:

(2) $$F_{total}=\sum_{i=1}^{m}J_{i}F_{i}^{R_{i}}\text{ Composition}$$

Then animation is a first-order ODE, with the time base generator γ(*t*) that induces terminal attractor dynamics (Zak, 1988) and the matrix *A* that plays the role of virtual admittance (the modulation of this matrix affects the relative degree of participation of the different joints to the common synergy):

(3) $$\frac{d\vec{s}}{dt}=\gamma (t)A\cdot F_{total}\text{ Animation}$$

We may assume that a symbiotic robot has implemented this kind of body schema and is also able to duplicate it for anticipating/interpreting the movements of its partner.

As an example, let us consider the task of whole-body reaching, that implies two concurrent sub-tasks: (a) reaching a target; and (b) maintaining the vertical projection of the center of mass inside the support base (Morasso et al., 2010). The animation of the body schema, in order to generate a stream of motions consistent with the double-task above can be carried out by activating five different force-fields: (1) a *Focal field*, applied to the hand and pulling it to the designated target; (2) a *Postural field*, applied to the pelvis, that aims at keeping the projection of the CoM inside the support base; (3) a *Range of Motion (ROM) field*, applied to each joint in order to repulse joint angles from the physiological joint limits; (4) *Head gaze field*, that aims at keeping the gaze directed to the target by inducing appropriate rotations of the cervical joint; and (5) *Neck field*, that stabilizes the neck, by attempting to keep it approximately vertical (see Figure 3 for a simulation).

It is important to note that the synergy formation process illustrated above is not a motor control mechanism in the strict sense and, in particular, is not directly responsible for muscle activation. Its function is to provide a reference body image (generated in virtual time) that satisfies a number of criteria (approaching the target, maintaining “static” stability, keeping rotations inside the RoM, looking at the target, and stabilizing the neck) in such a way to allow feedforward, feedback and stiffness control mechanisms to be activated in real-time in an optimal manner.

The use of force-field methods for solving problems of synergy formation and path planning has a long history in robotics (Quinlan and Khatib, 1993; Brock and Khatib, 1997; Khatib et al., 1999), although the force fields are intended to implement specific heuristics, e.g., for path planning, rather than as the “heart” of a general body schema. Early robotic research soon recognized that the basic capabilities needed to enable robots to operate in human-populated environments include an assistance ability they can bring to humans in performing various physical tasks. Typical operations are composed of various tasks, some of which are sufficiently structured to be autonomously performed by a robotic system, while many others require skills that can only be executed by a human worker. In particular, the investigation of a framework to integrate real-time collision avoidance capabilities with a global collision-free path has resulted in the *elastic band* *approach* (Quinlan and Khatib, 1993), which combines the benefits of global planning and reactive systems in the execution of motion tasks. The concept of elastic bands was also extended to nonholonomic robots producing the *elastic strip concept* (Brock and Khatib, 1997), which allows the robot’s free space to be computed and represented directly in its workspace rather than in its high-dimensional configuration space. A related major issue for interactive robots is safety. Any collision involving the robot and the human must result in a soft bouncing of the robot. This is called compliant behavior. It is a common approach to apply impedance control to give interactive robots a compliant behavior, with the drawback of reducing precision. An alternative is provided by a control scheme in which two control modes are embedded (Erden and Tomiyama, 2010): in the first mode, the robot maintains a reference position with given impedance characteristics; in the second mode, the robot follows the intention of the human by updating the reference position. The switching between the modes is again maintained by human intervention using no physical switch or sensor: indeed, the robot can detect the human intent by observing the impact of the physical contact on the control effort.

In sum, the body schema animation framework offers: (a) *Computational simplicity—*circumventing the need for explicit kinematic inversions while shaping motor output for action generation; (b) *Goal specific configurability* to coordinate diverse body and body+tool networks at runtime; and (c) *Ecological efficiency*, in the sense of recycling the same computational machinery for covert simulation of the consequences/goals of potential actions of one self and the other. We believe this is an essential building block for human robot symbiosis, well grounded on emerging trends from motor neurosciences and embodied cognition.

## An Imitation Machinery

The imitation machinery is a basic block of social cognition that requires computational mechanisms for the detection of the motor intentions of the partner, a shared memory system for the accumulation of knowledge, a set of physical interaction primitives for exchanging critical cues during cooperation, as well an animated body-schema for actual synergy formation. Imitation is an advanced behavior whereby an individual observes and replicates another’s behavior, thus carrying out an important function of social communication, learning and training (Ellwood, 1901). Imitation involves movement and multimodal perception (visual, auditory, haptic). From the neurological point of view the existence of some kind of “imitation systems” in the brain was suggested time ago by Liepmann (1908) during his study of the cerebral localization of functions that lead to the characterization of different forms of apraxia. More recently, human brain imaging studies revealed a network of regions in the inferior frontal cortex and inferior parietal cortex which are typically activated during imitation tasks (Iacoboni et al., 1999) and it is likely that such network is closely related to the so called mirror neurons system (Rizzolatti and Craighero, 2004; Oztop et al., 2006; Rizzolatti and Sinigaglia, 2010), although other brain areas might be involved as well (Kilner, 2011) and the actual role of mirror neurons in action understanding is still subject to controversy: consider, for example, Hickok (2014) and Rizzolatti and Sinigaglia (2015) for debating the two sides of the controversy. According to Jacob and Jeannerod (2005), the mirror system is the mechanism whereby an observer understands a perceived action of a partner by simulating, without executing, the observed sequence of movements. As a consequence, if we believe that such silent simulation of movements is indeed the animation of the body schema, then the mirror system must be linked to the body schema and the related simulation mechanism.

The study of social learning in robotics has been motivated by both scientific interest in the learning process and practical desires to design robots with suitable capabilities of collaboration with humans (Breazeal and Scassellati, 2002; Breazeal, 2009). This requires to address two fundamental problems: (1) a communication problem, via an appropriate language, in order to make explicit what to imitate and when starting imitation; and (2) a multi-sensory mapping problem, in order to allow the robot to map the perceptually observed demonstration onto its own action repertoire and back.

From the point of view of human-robot symbiosis, it seems quite reasonable that the robot’s cognitive architecture should include a working body schema of its own body and of its human co-worker and the corresponding mirror system. This is the prerequisite for learning by imitation and for effective cooperation. However, we should never forget that, in its generality, imitation is a very complex process that cannot be trivialized to the pure playback of a passively instructed set of motions: a practical example is given by so called “playback robots,” namely robots that repeat the same sequence of motions first instructed by an operator who puts the robot through a passive mobilization of a required sequence. We should consider that the pure playback paradigm lacks four basic requirements for the acquisition of motor skills: (1) generalization; (2) scale invariance; (3) motor equivalence; and (4) motor knowledge reuse across different skills. In contrast, an effective imitation process should allow the imitating robot to acquire a general kind of knowledge, easily adaptable to varying circumstances.

Imitation starts with the observation of the partner’s purposive action or skill through sensory channels (Chaminade et al., 2008). Although most research has been focused on the visual channel, in real life also the haptic channel is likely to play a role as in skill acquisition and skill training where the expert master (an artisan as well as a surgeon) shows the different aspects of skilled performance and may also intervene physically by gentle guidance. Moreover, imitation also requires that the two cooperating partners share a basic set of cognitive primitives that are the building blocks of the purposive action to be imitated. From the developmental point of view, it may be useful, for a symbiotic robot, to take into account the concept of zone of proximal development formulated by Vygotsky (1978, p.86) as “the distance between the actual developmental level as determined by independent problem solving and the level of potential development as determined through problem-solving under adult guidance, or in collaboration with more capable peers.” In Vygotsky’s opinion, when a student is inside the reachable zone of development for a particular task, providing the appropriate assistance will “boost” the capacity of the student to achieve the task in an efficient manner. In other words, imitation and execution are concurrent processes intrinsically framed in a social context. The imitation process can be conceived as a loop that combines three crucial streams of learning: (1) motor babbling (self-exploration); (2) imitative action learning (social interaction, observation); and (3) mental simulation. These are necessary prerequisites in order to give rise to sensorimotor knowledge that is endowed with seamless compositionality, generalization capability and body-effectors/task independence.

A previous study addressed in particular the task of teaching a humanoid robot to draw “Shapes” (Mohan et al., 2011) such as cursive script or graphical sketches. In order to bootstrap the teaching/learning process we must assume that the human teacher and the robot learner share a finite vocabulary of primitive shapes or graphemes and similar mechanisms for analysis/synthesis of complex shapes in terms of the primitive shapes. The quoted article uses a set of 12 primitive shapes, sufficient to characterize the overall shape of any line diagram in general, which were proposed by Chakravarthy and Kompella (2003) in the framework of catastrophe theory. The imitation loop is then formulated by integrating a number of transformations applied to representations expressed in different reference frames, namely the abstract frame of shapes, the egocentric frame of the shapes as seen through the robot’s eyes, the end-effector frame attached to the robot-grasped pen, and the motor frame related to the robot’s body schema. As a consequence, the imitation machinery is characterized by the same general feature of multi-referentiality that was already mentioned as a key element of the animated body schema. Mental simulations and real simulations are indeed applied to the robot’s body schema in the course of this process, thus emphasizing the multireferential nature of the imitation loop. Figure 4 shows one of the final snaps of the imitation process where the iCub robot (Metta et al., 2010) learns to write a demonstrated shape. A notable feature is that the skill learning is cumulative and open-ended, i.e., initially starting with demonstrations simple shapes (for example, “C, U, I,” etc.), the learnt motor knowledge subsequently exploited while swiftly learning/reproducing composite shapes (for example, “S, O,” etc.) and then perfected while learning to use diverse tools as an extension to the body (Mohan and Morasso, 2011). The computational framework also suggests that the human ability to almost spontaneously imitate an action with a good enough prototype is based on recall from the procedural memory of the motor knowledge related to previously learnt skills that are structurally similar to the present observed action.

In the present example (Figure 4), the imitating robot focuses only on the hand trajectory of the teacher, ignoring his whole-body movement. However, it is quite easy to outline interaction paradigms where both end-effector and whole-body movements matter. For example, suppose that the human teacher is a master of Tai Chi Chuan and iCub is learning the sequence, in its generality not in a pure playback fashion. Of course, the roles of the two partners can be switched: a robot Tai Chi master teaching a human pupil. In any case, this imitation paradigm can be formulated by hypothesizing that the pupil operates with two body schemas: one for performing mental/real simulations of his movements and another that approximates progressively the movements of the partner. Ultimately, the two body schemas will converge to produce the same movement patterns.

A second extension of the imitation process, to be implemented in a future generation of symbiotic robots, is a paradigm where the teacher interacts with the learner not only through the exteroceptive channels (vision and audition) but also via haptic interaction, namely by providing assistive or resistive force fields to crucial body parts of the learner. This kind of haptic interaction must be of sufficiently small amplitude and/or applied intermittently in time in order to avoid the passive movements that occur in playback robots and that are totally detrimental for achieving skilled behavior. The crucial role of haptic interaction in the imitation game is the capability of the pupil to transform the physical force field, carefully applied by the master, into a virtual force field to be integrated (and generalized) in the internal representation of the task.

## Motor Intentions Machinery and Body Language

A motor intention machinery is also a fundamental block of social cognition, grounded on multimodal observation of the partners as well as expectations retrieved from a shared memory system. In general, we agree with (Jacob and Jeannerod, 2005) that an action is a goal-directed sequence of bodily movements initiated, monitored, and fine-tuned by what may be called a “motor intention.” More specifically, by considering the distinction (Pacherie, 2000) between *basic actions*, i.e., actions aimed at the achievement of an intermediate/immediate goal, and *non-basic actions*, whose goal is farther away in space and time, a motor intention in the strict sense should be limited to the decision to perform a basic action.

From the point of view of human-robot symbiosis, the capability of reading the motor intentions of a partner in an implicit, non-verbal manner is an essential requirement (Sandini and Morasso, 2018). Detection of motor intentions to act can be carried out in a reliable manner by different techniques of analysis of brain activity (Gallivan et al., 2011; Haufe et al., 2011), however this is a too invasive method to be utilizable by symbiotic robots. On the other hand, the detection task is much more difficult at the behavioral level because it involves a broad range of evaluative processes including the decoding of biological motion, prior knowledge of object properties, and abilities for recognizing task space requirements and social contexts (Grafton, 2009). It is also becoming increasingly evident that some of this decoding is based in part on the simulation of other people’s behavior within our own nervous system, in agreement with the general principle of this article about the crucial role of an animated body schema for symbiotic human-robot collaboration.

There is now solid evidence from recent functional imaging studies that observation by one person of another who is engaged in a task is likely to induce in the observer a widespread, bilateral network of cortical brain regions in a highly reproducible manner (Grafton, 2009; Ptak et al., 2017). This distributed network is likely to support many subtasks, including the circular link of perceptions to action and action to perception, the simulation of observed movements in relationship to known movements, and the storage of physical knowledge (both of self, interacting partner, and manipulated objects/tools) that can be used for simulation. In other words, observation of others’ actions may induce a subliminal activation of motor pathways (motor resonance) that is mediated by fronto-parietal networks: this may include the mirror neuron system but also subcortical structures (de Gelder, 2006) in order to support some “hard-coded” reactive mechanisms necessary for rapid response during interaction. More generally, the attribution of intentions may be associated with the “theory or mind” network supported by the Temporo-Parietal Junction (Saxe and Kanwisher, 2003).

In order to support human-robot symbiotic interaction and collaboration, it is crucial for robots to establish a bidirectional communication channel with their human partners that includes some form of access to mental states. This entails both enabling robots to read the behavioral patterns of humans and to express their own intentions and internal states with their motion. This means that a robot should be able to read its partner’s mind and to express its own behavior in a form readable to a human observer. Extensive research has been conducted on both fronts (Dragan and Srinivasa, 2014; Mavridis, 2015), with a particular focus on the role of eyes, proposed as a proxy to convey and read mental states (Ruhland et al., 2015; Palinko et al., 2016). Recently, the attention has been directed toward the intuitive messages associated to body motion (Sciutti et al., 2012; Bisio et al., 2014) and to the design of computational models able to improve the legibility of motor intentions from the perspective of the action partner, including gaze and hand motions (Sciutti and Sandini, 2017; Figure 5). The goal is the development of a human-aware movement framework, which can produce safe, comfortable, socially acceptable, and readable motions, especially in the context of collaborative manipulation (Sisbot and Alami, 2012; Sciutti et al., 2013, 2014, 2015; Sciutti and Sandini, 2017). This is particularly important for humanoid robots since their actions can be processed similarly to human actions and, if so, should trigger a similar response in the human partners Sciutti et al. (2013, 2015). In other words, there is ground to believe that optimizing robotic movements to implicitly satisfy specific kinematic regularities (Casile et al., 2010) improves legibility of robot motions. This approach should generalize to different situations, different spatial configurations, or even different tasks (Busch et al., 2017), also taking into consideration how the regularities in kinematic patterns are mapped into the perceptual space of the observer (Vignolo et al., 2017), a sensory requirement for the legibility and exploitation of movement-based reciprocal communication.

In general, the prerequisite to move from fundamental principles of motor control to an application in robotics is hence making the rules extracted from human motion robust to the application in novel situations, something that is likely to require from the robots specific cognitive skills to be shared with the human partners. As a result, in addition to the above-mentioned ability to reveal its intentions and to read the mind of a partner through covert motion signals, a robot should also be able to exploit voluntary gestures and body language, as components of communication that can be linked to language in general (Sciutti et al., 2018). As a matter of fact, the concept of “gesture” includes a multiplicity of communicative movements of different parts of the body, with emphasis on hands and eyes. Often, gestures are assumed to comprise a channel distinct from language, but careful investigation challenges this traditional view: gestures and language should be thought of as a single system (McNeill, 2005). The study of gestures has grown significantly in recent years, revitalizing the link with de Jorio (1832), recognized as the first ethnographer of body language (see Kendon, 2000, who translated from Italian the book by de Jorio). In particular, McNeill and Levy (2009) proposed a classification scheme of gesticulations or speech-framed gestures based on four “dimensions”: “*iconic*” (for representing images of concrete entities and/or actions); “*metaphoric*” (for representing more abstract content); “*deictic*” (for locating entities and actions in space); “*beat*” (for signaling to a partner the temporal locus of something that is felt to be important with respect to the larger context).

We believe that this classification could be the starting point for defining a human-robot gesture language for supporting human-robot collaboration. It should not be meant of as an alternative to spoken language that indeed is an area of research and development in humanoid robotics. Spoken language covers a quite different area of communication with respect to gesticulation and body language: this is quite clear in human-human interaction and a fortiori it should hold even stronger in human-robot symbiosis. The underlying issue is also safety and robustness in human-robot implicit communication. This will require to implement in the robot motor controller the kinematic/dynamic invariants that are implicitly assumed by humans as characteristic of different kinds of motor intentions. Moreover, after having defined a suitable gesture language, as close as possible to established social standards, robot companions and human partners should be trained to use it in order to optimize symbiotic collaboration while minimizing mis-understanding. In other words, social robotics requires to define a social standard for well-educated human-robot symbiotic interaction, also taking into account what has been described as “vitality forms” of social communication (Di Cesare et al., 2018).

## Physical Interaction Paradigms for Skill Transfer

Physical human-robot interaction is usually associated with control rather than cognition. However, this is not the case if we focus on the wider horizon of symbiosis between a human and a robotic partner, bridging the gap between covert and overt actions on the basis of imitation paradigms and motor intention capabilities. As a matter of fact, physical interaction between human and robot cooperating for a common task is important at different levels for achieving and consolidating symbiosis, thus making the precise modulation of force a crucial communication channel. One level is *imitation*, thus extending the basic paradigm that is based on the visual/auditory channels: the teacher interacts with the naïve partner by providing assistive or resistive force fields to crucial body parts of the learner in order to suggest modification of his/her plan of action in critical aspects of a learning sequence. Another level is detection of *motor intentions*, namely physical suggestions of modification provided by an expert to a naïve partner. Such two aspects of physical interaction complement imitation and intentional processes by providing a stream of overt, real-time information that enhances covert, virtual-time paradigms based on internal simulations. The goal of all of this is supporting skill acquisition and skill transfer from an expert partner to a naïve partner by combining covert and overt actions.

The mechanism of overt interaction is captured by what is known as *shared control*. Shared robotic control, first described by Steele and Gillespie (2001), evolved from initial telerobotics applications: it is a form of haptic assistance in that the haptic robot contributes to execution of a dynamic manual task by a human partner via appropriately modulated force commands. In addition to the function of assistance, the shared-control approach offers a method for actively demonstrating desired motions during virtual environment interactions. In many applications of shared control, it has become clear that users of assistive devices prefer to cede only a minimum amount of control authority to the machine. Thus, if we consider the overall spectrum of shared control configurations we may locate, at one end, full manual control (i.e., direct teleoperation), and at the other full robot control: in between lies a continuum of shared control paradigms, that blend—whether by fusion or arbitration—the inputs from human and robot control. As a consequence, the design problem for human-robot shared control paradigms, is to find a sweet spot along this continuum that optimizes performance, effort, reliability etc. as a function of any specific task. Different approaches have been investigated in this context. For example, Dragan and Srinivasa (2013) proposed that rather than simply executing the human user’s input, which may be hindered by the inadequacies of the interface, the robot assistant should attempt to predict the user’s intent and softly assist him in accomplishing it, according to an intuitive formalism that captures assistance as policy blending. In shared control, the strength of assistance, also referred to as assistance level, is one of the main design factors. While many existing implementations mainly realize fixed assistance levels, it has been shown (Passenberg et al., 2013) that adaptive assistance policies can outperform constant assistance policies and switching assistance policies have advantages over continuously adapting policies (Oguz et al., 2010). It is also easy to realize that the issue of *switching assistance*, mentioned above, is deeply related to the issue of *intermittent control*, which challenges the common view that the brain continuously controls the commands to the muscles for executing what appear as a smooth flow of actions. In contrast, there are reasons for believing that motor commands are produced discretely, in an event-driven, asynchronous manner rather than the continuous-synchronous manner of conventional control paradigms (Bottaro et al., 2005, 2008; Asai et al., 2009; Gawthrop et al., 2011). The advantage of intermittent control paradigms, in terms of robustness of the control scheme, is particularly evident in feedback control problems where the feedback delay is comparable to the intrinsic time constants of the controlled process, for example in the stabilization of the upright standing posture or a hand-held inverted pendulum. The idea is that a simple observer of the dynamic state should evaluate the degree of danger, for example in terms of stability, and switch on feedback control only when the degree of danger overcomes a safety threshold. In this case, the orbit of the system, when the control is switched off, would follow the intrinsic dynamics of the body thus allowing the brain, among other things, to continuously update the internal model of its body.

In any case, basic perception–action links as in haptic interaction are crucial for many social interaction paradigms (Wolpert et al., 2003; Knoblich and Sebanz, 2006). In particular, haptically linked human dyads have been studied by many authors in recent years (Reed et al., 2006; Reed and Peshkin, 2008; van der Wel et al., 2011; Groten et al., 2013; Ganesh et al., 2014). Generally speaking, it turns out that “two is better than one” (Masumoto and Inui, 2013; Ganesh et al., 2014). For example, the analysis of task completion times in haptically linked dyads, indicated that dyads performed significantly faster than individuals, even though dyad members exerted large task-irrelevant forces in opposition to one another, and despite many participants’ perceptions that their partner was an impediment (Reed et al., 2006). It was also shown (van der Wel et al., 2011) that haptic coupling allows dyads to amplify their forces to generate a haptic information channel. Moreover, there is evidence (Groten et al., 2013) that a haptic channel is quite effective in facilitating intention integration between the two members of a dyad. The natural character of haptic interaction in a human-robot dyad is also suggested by a kind of “Haptic Turing Test,” (Reed and Peshkin, 2008) which showed that human participants consciously and incorrectly believed their robotic partner was indeed human.

Having demonstrated that physical coupling between two subjects may be advantageous in joint tasks, the next step for better understanding how to improve the symbiosis of a human-robot dyad is to investigate how two partners mutually exchange information to exploit the coupling in order to facilitate the transfer of skilled know-how from an expert performer to a naïve user, as in vocational training (Akshay et al., 2013). For this purpose Avila Mireles et al. (2017) adopted a reversed, novel perspective to the standard one that focused on the ability of physically coupled subjects to adapt to cooperative contexts that require negotiating a common plan. The study investigated how training in pairs on a novel task affects the development of motor skills of each of the interacting partners. The task involved reaching movements in an unstable dynamic environment using a bilateral non-linear elastic tool that could be used bimanually or dyadically (Figure 6). The main result was that training with an expert leads to the greatest performance in the joint task. However, the performance in the individual test was strongly affected by the initial skill level of the partner. Moreover, practicing with a peer rather than an expert appears to be more advantageous for a naïve user and motor skills can be transferred to a bimanual context, after training with an expert, only if the non-expert subject had prior experience of the dynamics of the novel task. The underlying issue is to find the optimal trade-off between *exploration* and *exploitation*: curiosity-driven exploration of the unknown dynamics of the task at hand by the novice, accepting low performance levels, vs. exploitation of the assisting action of the expert that may improve performance but also reduce the chance of the novice to experience a wide-range of dynamic contingencies, crucial for generalization and for a robust consolidation of the acquired skill. In general, such preliminary experiments point out at the circularity of the interacting process that may facilitate the development of growing levels of human-robot symbiosis.

## A Shared Memory System for Incremental Symbiotic Development

A shared memory system is a fundamental building block of social cognition if we look at human-robot symbiosis on a wider and longer time horizon, for example as a durable partnership in natural living spaces (work, social, etc.). In this case, the gradual accumulation and consolidation of diverse learning experiences (skills, casual relations, social relations) into the robots episodic memory becomes a crucial aspect of symbiosis and a powerful amplifier of the imitation machinery and the motor intention machinery. The critical advantage is that partial cues from the present environment like perceived objects, observed actions of others can then trigger associative recall of context specific past experiences that could be exploited for both goal directed reasoning and joint goal collaboration with other agents (Figure 7). This process of cumulative accumulation of experiences and knowledge into episodic memory could also be considered as the emergence of a preliminary form of consciousness in a symbiotic robot. The reason is that ultimately the functional utility of consciousness is to enable an agent (human/robot) not only to “experience the present” but also “re-experience the past” and “pre-experience” the potential future. Further, re-experiencing the past is directly linked to prospectively “pre-experiencing” future as also suggested by emerging trends from neurosciences like the discovery the Default Mode Network (DMN) in the brain (Raichle et al., 2001; Buckner and Carroll, 2007; Buckner et al., 2008; Bressler and Menon, 2010; Welberg, 2012; Suddendorf, 2013), known to be involved in constructive episodic simulation. Undoubtedly, this is a fundamental desirable feature if robots are to truly become our “companions” in unstructured natural living spaces.

Unlike in synthetic systems where memory is usually treated as a passive storage structure, in the domain of cognitive robotics modeling the implementation of episodic memory functions has recently been a topic of emerging interest (see, Vernon et al., 2015a for a review). Robot episodic memories have been instantiated both sub-symbolically, through auto associative networks (Mohan et al., 2014; Bhat et al., 2016) or symbolically, using content-addressable image databases with traditional image indexing and recall algorithms (Tenorth and Beetz, 2012; Tenorth et al., 2013). As a side effect, since such repertoire of episodic memories are derived from direct experiences (of the robot—see Figure 7), it also finesses the symbol grounding problem (Harnad, 1990). Furthermore, the present state of the art in relation to episodic memory systems (Natural/Artificial) prompts further advancements in several directions that lie at the intersection of cognitive robotics, neurosciences and end user applications, mainly:

1. *Multimodal Recall* of past experiences triggered through multimodal cues from the present environment that requires both formulation of multimodal similarity index (as real worlds are never identical) and coupled retrieval dynamics with pattern completion properties (Hopfield, 2008).
2. *Cumulative “encoding and memory reorganization”* so as to facilitate recalled past experiences to be seamlessly combined with new actions, to learn something further, from new memories. A related issue is to avoid an explosive accumulation of memories by enabling forgetting through a survival of the fittest like competition mechanism for episodic memories (Mohan et al., 2014).
3. *Gradual Consolidation of episodic experiences* into the semantic memory thereby extracting causal invariances from rich sensorimotor experiences. Such generalization mechanism is also a core feature to facilitate reasoning by analogy, thus moving from object-action to property-action in relation to affordances, given the distributed property specific organization of semantic memory in the brain (Patterson et al., 2007; Martin, 2009).
4. *Memory to Combinatorial Creativity* that enables multiple past experiences to be recombined in novel ways. This is a natural consequence of both the constructive and rather imperfect nature of memory recall. Recent results suggest that overlaps between experiences enable the re-combination from initial state to a future simulated and desired state (Zeithamova et al., 2012; Mohan et al., 2014). This mechanism in a computational sense can be considered quite similar to path planning (in time) and is relevant also in the context of ongoing debate in the field of neurosciences to reconcile declarative memory and spatial navigation functions of the medial temporal lobe (Eichenbaum, 2014).
5. *Memory Clouds*: Beyond advancements in cognitive robotics and neurosciences (i.e., 1–4), and the alleviation of the grounding problem, a further advantage is that such artificial episodic-semantic memory systems also renders the knowledge contained inherently transferrable to other agents, provided their sensory-motor systems are compatible and there is a known mapping—direct or indirect—between the embodiments (Waibel et al., 2011), opening up the potential for significant advancement in the domain of cloud robotics, robot-robot, human-robot joint goal collaboration.

In sum, the ability to infer others goals by *looking* is based on *remembering* what we have learnt by *doing*. In this context, a growing memory architecture is a crucial building block for human robot symbiosis, introducing an element of “free choice” of what is worth remembering and/or learning.

## Conclusion

The next generation of robot companions or robot working partners will need to satisfy social requirements somehow similar to the famous laws of robotics envisaged by Isaac Asimov time ago (Asimov, 1942). The necessary technology has almost reached the required level, including sensors and actuators, but the cognitive organization is still in its infancy and is only partially supported by the current understanding of brain cognitive processes. The brain of symbiotic robots will certainly not be a “positronic” replica of the human brain: probably, the greatest part of it will be a set of interacting computational processes running in the cloud. In this article we review a small set of such computational processes or building blocks of a cognitive architecture shared by human and robot partner, that may constitute a preliminary attempt to give symbiotic capabilities to cobots of the next decades: (1) an animated body-schema; (2) an imitation machinery; (3) a motor intentions machinery; (4) a set of physical interaction mechanisms; and (5) a shared memory system for incremental symbiotic development. For each of such building blocks we investigated early possible computational formulations which are briefly reported in the previous sections of the article.

We would like to stress that our approach is totally un-hierarchical. As exemplified in Figure 8, the five building blocks of the shared cognitive architecture are fully bi-directionally connected. For example, imitation and intentional processes require the “services” of the animated body schema which, on the other hand, can run its simulations if appropriately prompted by imitation and/or intention, with or without physical interaction. Successful experiences can leave a trace in the shared memory system and chunks of memory fragment may compete to participate to novel cooperative actions, and so on and so forth. At the heart of the system is lifelong training and learning but, different from the conventional learning paradigms in neural networks, where learning is somehow passively imposed by an external agent, in symbiotic robots there is an element of free choice of what is worth learning, driven by the interaction between the robot and the human partner. The proposed set of building blocks is certainly a rough approximation of what is needed by symbiotic robots but we believe it is a useful starting point for building a computational framework.

Our approach is fully compatible with the embodied cognition point of view. At the core the proposed framework is also the concept of functional recycling or reuse of the same computational machinery in different contexts, for example generating actions, simulating actions and understanding others actions. The integrated framework (Figure 8) emphasizes that perspective that brain achieves its diversity of cognitive functions by *recycling* the same regions in a variety of circumstances, putting them together in different patterns of “goal” oriented functional cooperation: to facilitate the survival of a complex body in a highly unstructured and social world. In this context, looking at cognition not only as brain function but, brain-body-environment function, cognitive robots are valuable research tools to reenact the interplay between multiple “sensory, motor and cognitive” functions from the perspective of an integrated system in order to look for underlying computational basis.

In particular, we believe that a human or humanoid agent is a kind of *Monad* (in Leibniz’s sense) which includes three, non-separable elements: brain, body, and environment. Purposive behavior emerges from the dynamic interaction of the three elements. Thus, we can conceive a *Human Monad* and a *Robot Monad* when a human agent or robot agent operates alone for achieving a goal: as monads, the two agents may have completely different bodies and cognitive architectures. However, when the two agents interact in order to cooperate in a common task they must share at least the building blocks of the cognitive architecture. This idea is illustrated in a simple manner by Figure 9: the two monads are “fused” as two cells in a functional tissue. The brain+body of each agent becomes a kind of virtual environment of the other agent and both agents interact with and share the dynamics of the common physical environment.

It is clear that the building blocks of social cognition outlined above for human robot symbiosis are only a rough sketch of a potential architecture, with a number of open questions and big challenges both at the scientific and technological levels. In particular, it will be necessary to further investigate the computational organization of the building blocks and the communication paradigms among them, with the possibility to add new modules, e.g., a block for affective interaction, an ethics layer inspired by simulation theory of cognition (see, Vanderelst and Winfield, 2018) in contrast to symbolic rule-based systems. The full desiderata for developmental cognitive architectures (see, Vernon et al., 2016 for a review) includes several features value systems, attention, constitutive autonomy that are not fully dealt with in the present framework. From the technical/implementation point of view there is the huge challenge to encapsulate the building blocks outlined in this review article in an open software/firmware/communication infrastructure that makes possible the integrated architecture. The starting point is one of the main open source robot platforms that have been developed in the last decade, becoming a* de facto* standard, such as YARP (Metta et al., 2006; Fitzpatrick et al., 2008, 2014) or ROS (Quigley et al., 2007, 2009). Finally, only realistic testing of architectures for social intelligence and human robot symbiosis in unstructured natural living spaces (e.g. Hospitals, Shopping malls etc.) will enable the understating of advantages/weaknesses and foster innovation towards development of socially cognitive robots-working with and for humans.

## Author Contributions

GS, PM, AS and VM have contributed equally to the article.

## Conflict of Interest Statement

The authors declare that the research was conducted in the absence of any commercial or financial relationships that could be construed as a potential conflict of interest. The handling Editor and reviewer ACK declared their involvement as co-editors in the Research Topic, and their shared affiliation.
